# 

**Published:** 1875-04

**Authors:** 


☞ All communications other than of a business character must be addressed to Dr. Thos. S. Powell, 82 Pryor street.

☞ Write your name, post-office, county and State *plainly*. Be sure to say to which post-office you wish the RECORD sent, and always date your letters. We receive many well-written letters, but doubt even the authors being able to decipher their signatures.

☞ Send money by Check, Postal Order or Registered Letter. We are willing to endorse for the honesty of our subscribers, but cannot be responsible for the irregularity of the mails.

☞ We hope our friends, one and all, will rally to the support of the RECORD. Send the publishers three dollars, the subscription fee, and at least one new subscriber. Send to the principal editor your communications, plainly and carefully written on only one side of the paper.

☞ All due for 1873 and 1874 can be directed to Dr. T. S. Powell, or Powell & Goldsmith, as heretofore.

☞ We shall print several hundred extra copies of the January and February numbers of the RECORD, that all new subscribers who send in their names between this and the tenth of March, may have the volume for 1875 complete. Be certain to be in time.

### LIST OF CASH PAYMENTS SINCE FEBRUARY NUMBER.

| | | | |
|---|---|---|---|
| Dr. M. Giesy, Oregon | $3 00 | Dr. W. P. Anderson, Georgia | $3 00 |
| Dr. C. P. Sanders, Alabama | 3 00 | Dr. G. H. Thompson, Alabama | 3 00 |
| Dr. Louis Hadden, Louisiana | 3 00 | Dr. C. O. Nicholds, Arkansas | 50 |
| Dr. J. S. Bacon, Louisiana | 3 00 | Dr. J. E. Pope, Georgia | 3 00 |
| Dr. C. C. Hart, Georgia | 3 00 | Dr. S. T. Birdsong, Mississippi | 3 00 |
| Dr. L. W. Mobley, Georgia | 5 50 | Dr. G. W. Wimbish, Texas | 1 50 |
| Dr. A. A. Lyon, Mississippi | 3 00 | Dr. Z. T. Young, Louisiana | 3 00 |

### COLLABORATORS AND CONTRIBUTORS.

GEORGIA.

James A. Long, M.D.
W. L. M. Harris, M.D.
H. L Battle, M.D.
W. C. Musgrove, M D.
L. B. Bouchelle, M.D.
Thomas Burdell, M.D.
E. H. W. Hunter, M.D.
V. S Hitt, M.D.
J. E. Pope, M.D.
J. C. Wisdom, M.D.
W. W. Leake, M.D.
S. G. White, M.D.
W. H. Hall, M.D.
G. D. Case, M.D.
W. T. Grant, M.D.
J. M. Tules, M.D.
J. N. Cheney, M.D.
T. M. Shaw, M.D.
T. S. Mitchell, M.D.
H. B. Lee, M.D.
C. B. Nottingham, M.D.
W. L. Kirkpatrick, M.D.

KENTUCKY.

W. S. Taylor, M.D.
B. W. Stone, M.D.
Charles Kearns, M.D.

TEXAS.

S. M. Welch, M.D.

T. J. Heard, M.D.

L. R. Lincecum, M.D.

TENNESSEE.

J. D. Tucker, M.D.
F. A. Ramsey, M.D.
J. W. Hayne, M.D.
F. K. Bailey, M.D.

WEST VIRGINIA.

N. D. Tobey, M.D.

ALABAMA.

J. C. Knox, M.D.
Geo. F. Taylor, M.D.
J. B. Barnett, M.D.
S. M. Hogan, M.D.
J. T. Searcy, M.D.
A. C. Edwards, M.D.
M. W. Morton, M.D.
J. D. Rush, M.D.
J. J. Grace, M.D.

NORTH CAROLINA.

J. B. Hughes, M.D.
S. S. Satchwell, M.D.
A. B. Pierce, M.D.
H. Kelly, M.D.
Geo. A. Foote, M.D.
E. A. Anderson, M.D.
H. T. Bahnson, M.D.
John W. Booth, M.D.
R. S. Hallsey, M.D.

SOUTH CAROLINA.

E. T. McSwain, M.D.
G. M. Rivers, M.D.

OHIO.

J. Q. A. Hudson, M.D.
C. H. Tidd, M.D.
J. C. Bishop, M.D.
W. P. Wells, M.D.

NEW YORK.

Ely Van DeWarker, M. D.
C. C. F. Gay, M.D.
J. S. Bailey, M.D.

COLORADO.

Wm. R. Whitehead, M.D.

VIRGINIA.

F. Horner, Jr., M.D.
R. J. Preston, M.D.
J. S. Wharton, M.D.
Sam'l P. Ripley, M.D.
E. A. Drewry, M.D.
Rob B. Tunstall, M.D.
W. F. Barr, M.D.
L. B. Anderson, M.D.
J. N. Upshur, M.D.
B. P. Reese, M.D.

MISSISSIPPI.

C. B. Gallaway, M.D.
Edward Lea, M.D.
S. V. D. Hill, M.D.
E. T. Henry, M.D.
T. P. Lockwood, M.D.
Robert Kells, M.D.
W. L. Lipscomb, M.D.
W. T. Beall, M.D.
A. G. Smythe, M.D.
J. M. Lewis, M.D.
J. H. Payne, M.D.
T. H. Mayo, M.D.
E. Cutter, M.D.

ARKANSAS.

A. D. Hodge, M.D.
J. K. Hodge, M.D.
A. W. Hobson, M.D.
C. D. Hodge, M.D.

INDIANA.

G. A. Dyer, M.D.
A. Patton, M.D.
Robert Robson, M.D.

ILLINOIS.

John H. Weir, M.D.

OREGON.

W. M. Cusick, M.D.

